# Variability in UK Body Donation Information: A Comparison of Bequeathal Information and Consent Forms With Recommendations for Standardization

**DOI:** 10.1002/ca.70053

**Published:** 2025-11-26

**Authors:** Janet A. C. Philp, Kat A. Sanders

**Affiliations:** ^1^ University of Edinburgh Edinburgh UK; ^2^ School of Medical Sciences, Faculty of Medicine and Health University of Sydney Sydney New South Wales Australia; ^3^ Hull York Medical School University of Hull Kingston upon Hull UK

## Abstract

The use of human donor bodies for anatomical examination in the United Kingdom is regulated by the Human Tissue Authority (England, Wales, and Northern Ireland) and His Majesty's Inspector of Anatomy for Scotland. This study aimed to assess the variability of information provided to body donors and the associated consent forms across UK anatomy institutions. A total of 24 consent forms and information booklets were collected from all body donation programs across the UK. Building on previous research, each document was assessed against a checklist covering general information about the donation process, purposes and locations of body use, consent requirements, disposition of remains, and accessibility. The analysis revealed significant heterogeneity in the information provided. The findings suggest a need for standardization of body donation information and consent forms to ensure they meet ethical requirements for informed consent and to improve accessibility and inclusivity. Recommendations include ensuring consistency between information provided and consent forms, requiring confirmation of reading the information, standardizing age limits and medical condition statements, providing clear information for next of kin, and ensuring ethical oversight by institutional committees. Further research is needed on donors' perspectives regarding specific aspects of the donation process. These recommendations are proposed to provide a more consistent approach to sharing information about body donation, ultimately suggesting the development of a single, collaboratively produced form and information pack to minimize customization (and thus omissions).

## 1 Introduction

The human body has been used as an aid to education for centuries (Ghosh 2015). In the United Kingdom (UK), the supply of human tissue changed from the unethical historical context where it was procured through grave robbing (Richardson 1988) or even murder (Philp 2022), progressing to one of altruistic donation. This option was created by the 1832 Anatomy Act which later developed into two acts that cover the UK: the Human Tissue Act (2004) (for England, Wales, and Northern Ireland) for whom the Human Tissue Authority (HTA) regulates work under the Act; and the Human Tissue (Scotland) Act (2006) specific to Scotland and overseen by His Majesty's Inspector of Anatomy (HMIA) for Scotland. Individuals in the UK wishing to donate their body need to provide consent for “anatomical examination.” This can be done through a declaration in their will (this requires specific wording to be used, and may not be accepted at all institutions) or, preferably, by completing the appropriate donor form from their local medical school prior to death. Unlike some other countries where next of kin (NOK) can decide to donate the body of a loved one, first person donation has been the only route for body donation in the UK since amendments to the legislation in 1984 (Anatomy Act 1984). Whilst it is permissible for UK institutions and companies to import human tissue for education, training, and research purposes, this falls outside the scope of this study which focuses on UK‐based donors. Before examining the donation process further, the definition of “consent” used throughout this paper must first be established.

While the two Acts covering the UK are broadly similar, they differ in one very important detail for the purposes of this review. When the legislation was being developed alongside the recommendations following the Alder Hey scandal (Hall 2001), the review board established by the Scottish Executive was “of the view that ‘consent’ as currently legally understood is inappropriate and misleading in the context of postmortem examination and removal and retention of organs” quoted in Brazier (Brazier 2002; Mclean 2001). This concern originated from the fact that the parental rights for the treatment of children are limited to those rights which are in the “best interest” of the child; a concept that fails to properly convey its intended meaning when applied in the context of the deceased. Based on these concerns, the Scottish legislation uses the term “authorisation” to cover donation of human tissue for all individuals, while the legislation covering the rest of the UK uses the term “consent.” To add to the confusion, Scottish “authorisation” is given by donors on a form entitled the “Common Consent Form.” Whichever term is used (consent or authorisation), the central feature is the entitlement to say “no” (Kim 2019) and it is in that context that the term “consent” is used throughout this paper. It is also important to note that consent must also be “informed.”

The concept of informed consent for medical procedures on the living can trace its origins to the early 1900s (Bazzano et al. 2021), and it became the social norm following the Nuremberg trials at the end of the Second World War (Schütz and Braswell 2023). How informed the consent for procedures that occur postmortem is, has been a subject of recent research (Keet et al. 2023; Farsides and Smith 2023; Smith et al. 2022; Johnson et al. 2023; Zealley et al. 2022).

In the United States (US), Zealley et al. (2022) found a wide range of information in donation guidance across 110 forms, leading to a question of whether the consent is sufficiently informed (Johnson et al. 2023; Zealley et al. 2022; Chung and Lehmann 2002). While many US states adopted the Uniform Anatomical Gift Act 1968 (and then 1987 and 2006), there are no regulatory or inspection bodies in place to ensure compliance and uniformity of good practice. In contrast, the UK regulatory bodies (the HTA and HMIA) each provide guidance as to what information should be included in bequeathal documents in the UK. It may therefore be expected that UK bequeathal information and consent forms should be fairly homogenous. This study aimed to establish if this is indeed the case, by examining the scale of variability of information provided to body donors, and the associated consent forms, by institutions that conduct anatomical examination in the UK.

## 2 Materials and Methods

Bequeathal documents (including consent forms and information booklets) were accessed from all body donation programs listed on the HTA website (*n* = 19), covering 27 medical schools in England, Wales, and Northern Ireland, as well as the national repository. In most cases, UK medical schools each run their own body donation program; however, in London and South East England, the London Anatomy Office centrally co‐ordinates body donation for nine medical schools which is why the number of forms is fewer than the number of medical schools. A further five forms were collected from medical schools in Scotland. In total, 24 consent forms and information booklets were gathered for analysis. This represents 100% of the medical schools listed on the HTA website as institutions that receive body donations within the UK. Many were downloaded from the schools' webpage on body donation. Some medical schools had to be contacted directly to email or mail the consent forms. All forms were originally collected in August 2023 and re‐retrieved and reviewed on August 15, 2025 to check for updates; data from the latter review is presented here. Since initial retrieval, one of these body donation programs has ceased operations and another has paused them, though they have both been included in this analysis.

A checklist was constructed based on the guidance from the American Association of Clinical Anatomists (2017) and previously published work by Zealley et al. (2022) on the information provided by body donation programs in the US. The checklist consisted of the same four sections established by Zealley et al. (2022) to allow direct comparison where relevant. Building on this, new items were included in some sections, plus a new fifth section that captured how accessible and inclusive the documents were. Each information pack and consent form was assessed against the full checklist (Table 1) by the lead author. The data recorded for all forms were then moderated by the second author to confirm the accuracy and consistency of the detail recorded. The extracted information was recorded in a spreadsheet with a simple yes/no response for all criteria, plus statements capturing additional details to inform the level of consistency between UK body donation programs.

**TABLE 1:** Information booklet and consent form checklist.

Section 1—General information about the body donation process	
Institution receiving the donation^a^	Is it clear what institution is receiving the donation
Donation time frame (including indefinite retention)^a^	Is the time frame of donor usage clear
Applicable fees, if any^a^	Are any fees clearly explained
	Whether there are any expenses (for the NOK/executor) associated with the donation and whether these are explained
Serology testing/disclosure of test results^a^	Is there any mention of serological tests and the sharing of results and incidental findings?
Medical records/information gathering/release practices^a^	Is there any mention of accessing medical records or medical history being collected?
Possibility of declining the donation during the registration process or time of death and the potential reasons for decline^a^	Is the possibility of declining the donation mentioned, and potential reasons why defined?
Information about what to do at time of death	Is there clear information for families and executors on what to do and what might happen after the donor has passed away?
Withdrawing consent	Is the process to withdraw consent described?
Confirmation or acknowledgement of consent/authorization receipt	Is there a formal confirmation that the completed consent form has been received by the receiving institution?

Section 2—How and where the body may be used	
Donation purposes/use (research/education/plastination/display/training)^a^	Are the purpose(s) to which the donation will be used clear?
End users of anatomical materials^a^	Are the end users of the donation identified?
Use location (national or international) and possibility of transfer^a^	Is there any mention of transfer of the donation to an alternative location
Images (acquisition and use)^a^	Is there any mention of taking images and for what purpose these images may be used?
Preparation methodologies (recovery, disarticulation, embalming, plastination, etc.)^a^	Is there any mention of the methodologies used in the preparation of the donor material
Institutional process to decide on other potential uses (e.g., research projects)	Is there a committee and/or formal decision‐making process to determine further use of a donation?

Section 3—Consent requirements	
Age and competency status of the donor^a^	Is there any mention of limits on the donor, e.g., age limits, competency
Witness signatures^a^	How many witnesses are required, and are there limits on who can be a witness?
Representative form	Is there a representative form (available or mentioned) for someone else to sign if the donor is physically unable to?

Section 4—Disposition of remains	
Method of disposition described, e.g., cremation, scattering, burial^a^	Are the method(s) of donor disposition specified?
Family attendance	Is there an option for the family to attend the cremation service?
Disposition of retained parts	If indefinite retention is consented, how are the retained parts eventually disposed of?
Designated person to whom remains should be released^a^	Is there an opportunity for the donor to specify who their remains (e.g., ashes) should be released to?

Section 5—Accessibility and inclusion	
Has a dedicated webpage for the body donation program	Are the contact details and other information related to the body donation program available on the home institution's website?
Ease of access to the document(s)	How are the document(s) obtained by potential donors (e.g., downloaded from the web, need to contact program and then sent)?
Large font or braille formats offered	Is the form available in alternative formats (e.g., Braille)?
Translation to other languages offered	Is the form available in other languages?
Record of updates to information and form	Is there a revision history and/or version number to indicate whether updates have been made?
Information and consent form as a single document	Are the information booklet and consent form physically (same envelope) or digitally (same file) linked?
Contact information for body donation program	Are the contact details of the department provided in the document? Can these details be searched for online, available on a donor card, etc.?
Gender‐neutral language	Are the terms used in the information and consent form gender neutral?

^a^ This item was used in the paper by Zealley et al. (2022).

**TABLE 2:** Tabulated results for Section 1: General information about the body donation process.

Item	Percentage (*n*)
Institution receiving the donation	100 (24)
Donation time frame	100 (24)
Applicable fees (for NOK/executor)	95.8 (23)
Serology testing	12.5 (3)
Medical records/information gathering/release	33.3 (8)
Possibility of declining donation	100 (24)
Information about what to do at time of death	95.8 (23)
Withdrawing consent	75 (18)
Confirmation of acknowledgement of consent receipt	25 (6)

**TABLE 3:** Tabulated results for Section 2: How and where the body may be used.

Item	Percentage (*n*)
Donation purposes/use (research/education/plastination/display/training)	100.0 (24)
End users of anatomical materials	79.2 (19)
Use location (national or international) and possibility of transfer	79.2 (19)
Images (acquisition and use)	87.5 (21)
Preparation methodologies (recovery, disarticulation, embalming, plastination, etc.)	126.7 (4)
Institutional process to decide on other potential uses (e.g., research projects)	29.2 (7)

**TABLE 4:** Tabulated results for Section 3: Consent requirements.

Item	Percentage (*n*)
Age and competency status of the donor	45.8 (11)
Witness signatures	100 (24)
Representative form	25 (6)

**TABLE 5:** Tabulated results for Section 4: Disposition of remains.

Item	Percentage (*n*)
Method of disposition described, e.g., cremation, scattering, burial	95.8 (23)
Family attendance	29.2 (7)
Disposition of retained parts	37.5 (9)
Designated person to whom remains should be released	16.7 (4)

**TABLE 6:** Tabulated results for Section 5: Accessibility and inclusion.

Item	Percentage (*n*)
Has a dedicated webpage for the body donation program	95.8 (23)
Ease of access	
Download document(s) direct from website	54.2 (13)
Contact (info on website) to be sent document(s)	41.7 (10)
Contact (info only on HTA website) to be sent document(s)	4.1 (1)
Large font or braille formats offered	29.2 (7)
Translation to other languages offered	25 (6)
Record of updates to information and (/or) form	54.2 (13)
Information and consent form presented as a single document	54.2 (13)
Contact information for body donation program	100 (24)
Gender‐neutral language	79.2 (19)

### 2.1 Checklist Criteria

Section 1 covered the general information about the body donation process shared on the forms. New items included providing information on how to withdraw consent, whether confirmation of receipt of a donor's consent form would be communicated to them, and information on what the next‐of‐kin or executor should do at the time of the donor's death to proceed with the donation of their body.

Section 2 included the purposes for which the body may be used, including where and by whom this would be done. In addition, information on whether there was a formal process or committee to decide specific potential uses (e.g., approval of research projects) within the bounds of the consent was recorded.

Section 3 looked at the requirements to accept a donor's consent. The Zealley et al. (2022) checklist stipulated there be two witness signatures, and this was the case for 94% of US donation forms. In the UK, the requirement is one witness, and this requirement is reflected in the checklist. Furthermore, a new item was added on the availability and use of a representative consent form (a template is provided by the HTA) if the donor is physically unable to sign but still has capacity.

Section 4 covered the disposition of the body at the end of the donation period. Two items were added to the checklist to record if a donor's family could attend the cremation service (where there was one), and in the case of retained parts, how these might eventually be disposed of.

Finally, Section 5 was a completely new section that recorded how the information and consent form could be accessed and how inclusive this was. This covered such information as whether receiving institutions had a dedicated body donation webpage and how information was able to be accessed (e.g., downloaded, email or phone request). It also recorded if the information booklet and consent form came as one document, or whether it was possible to obtain the consent form without the information booklet, and the number of pages of the combined information and consent form. Whether the information was available in different formats, including large print and Braille (Cabinet Office (Disability Unit) 2021) and translation to other languages was recorded. Use of gender‐neutral language throughout was also noted as per the National Health Service guide for inclusive content (2021). It was also noted whether there was a version number or date on the document, and if the contact details for the department were obvious in the information.

## 3 Results

### 3.1 Section 1—General Information About the Body Donation Process

In all the cases it was clear what institution the form belonged to (Table 2). One form, however, lacked the University logo from the front cover and two other forms displayed their university logo alongside the HTA logo. The HTA do not permit the use of their logo on consent forms to ensure it is clear that potential donors are not donating to the HTA, but to an institution. The presence of the HTA logo on some institutions' forms suggests that these were last compared alongside the HTA example donation form before 2018, when the HTA model documents were last updated and their logo removed from their example form.

All institutions provided a donation time frame; however, the clarity of this message was variable. For the Scottish medical schools, it was very clear that the time frame of the donation would be a maximum of 3 years, unless permission was given to retain parts. In the rest of the UK, there were options for indefinite retention of the whole body or disposition after 3 years. It varied between establishments as to whether parts could be retained indefinitely or, if the 3‐year option was selected, the disposition of the whole donor would be carried out after 3 years. The options on the form did not always align with the options in the information booklets, and in two instances the explanation of retained body parts did not match the explanation on the forms. These inconsistencies were found in the HTA‐based forms and were all around the retention of body parts for longer than retention of the whole body. For example, the information for one medical school explains:

It can be useful to retain parts of your body after the examination is complete and the rest of the body has been cremated or returned to your family's undertaker for a private burial. If you would prefer the funeral to take place within three years but are happy for us to keep such samples, please tick option 1 on the form.

However, option 1 on the accompanying form reads:

I do not place any restrictions on the length of time my body or body parts may be retained.

This would allow the whole body to be retained indefinitely, which is not in line with the explanation in the booklet.

Fees were mentioned in multiple ways in the information provided. Almost all programs mentioned that transportation fees would be incurred if the donor died outside of the establishment's catchment area and that there would be fees if the family wanted a private funeral (*n* = 23). One program mentioned fees associated with the recycling of metal parts and another mentioned checking prepaid funeral plans as to what financial impact would occur as a result of donation. Six information packs stated that if they ran courses for which they charged fees then these fees were only to cover costs, and no profit was made from donations. One implied there was a fee for the family to attend the funeral of their relative.

Only eight stated that medical records would be accessed, with three of these asking for permission. Similarly, seven forms (from different institutions) had a space for the donor to self‐report their own medical history, usually with a focus on surgeries. Eight information packs mentioned that there would be a discussion with a medical practitioner to decide on the suitability of the donation based on the cause of death. Only three establishments stated that there would be tests conducted to protect their staff from infectious disease, and these results would not be released to the family. This low number is congruent with the Zealley et al. (2022) from the US where only 8% of donation documents included this information. In the UK, it is perhaps reflective of local regulatory requirements, as donor testing is only indicated when specifically importing fresh frozen donor bodies (point 69, HTA Code of Practice C for Anatomical Examination 2023) which is done by very few UK institutions. In contrast, serology testing is more common in Australia and New Zealand (Jenkin and Keay 2025) where regulatory bodies more explicitly outline how the NOK of potential donors should be informed of screening tests and any result‐dependent outcomes (NSW Health 2023).

Every form mentioned the possibility that the donation may not be accepted; however, the reasons for declining a bequest were extensive. A total of 130 medical conditions that would affect the ability to donate were listed across the forms. The majority of these were conditions that may not have been present at the time of consent, and the use of jargon and technical language was common. The severity of a condition varied across forms; for example, “jaundice,” “severe jaundice,” and “jaundice of an infectious origin” were all listed as potential reasons to decline a bequest. The number of limbs that could be amputated and a donation still being acceptable also varied. BMI or variations in weight, either below or above the normal range, were mentioned along with the requirement for a postmortem or donation of organs. Most forms mentioned the possibility of declining donations during periods when the department was closed (e.g., national holidays) or if they did not have the capacity to accept the donation.

The information for families and what to do at the time of the donor's death was usually embedded within the documents (*n* = 23). A few institutions had it as a clearly labeled separate section, and one establishment had it as a section that could be filed separately by the NOK or the executor. Some forms worded this section as though addressing it to the donor, when addressing the NOK or the executor would be more appropriate.

Only six programs confirmed they would acknowledge receipt of the forms and three of these required a stamped addressed envelope to do so. Three‐quarters of information packs (*n* = 18) informed potential donors that they can withdraw their consent and how to go about this.

### 3.2 Section 2—How and Where the Body May Be Used

When describing the purpose of the donation there was very little variation from the recommended text specifically on the consent forms (Table 3). All the Scottish forms contained the wording “I wish that after my death my body be made available for anatomical teaching, training and research within the guidelines of the Anatomy Act.” Within the rest of the UK, forms used the standard sentence “I wish to donate my body after my death. I understand that it may be used for anatomical examination, education or training relating to human health, and research in connection with disorders, or the functioning, of the human body.” A couple of information booklets had paragraphs explaining what anatomical examination meant and giving examples of the research for which donors may be used. One form was very specific and mentioned the various locations where the donors may be used.

Four fifths (*n* = 19) contained some information about the end users of the donation. The forms listed medical students, allied health care professionals, science students, and anatomical artists but there was also mention of external courses and any other group that the Designated Individual (each HTA‐regulated institution has a Designated Individual who is responsible for scheduled activities carried out in that institution) felt appropriate. Five establishments made no mention of who would use the donation.

Most (*n* = 19) of the forms mention the possibility of transfer to other medical schools. Once again, there was consistent provision of this information by all the Scottish establishments (*n* = 5). In contrast, only 73.6% (14 out of 19) of forms from the rest of the UK mentioned the possibility of transferring the donation to another location, though some went a step further and included transfer as a specific consent item to tick on the form itself.

In alignment with International Federation for Anatomy Associations published recommendations (Cornwall et al. 2024), 87.5% of the consent forms have a specific tick box related to consent for imaging, with some drawing attention to the fact that this was not covered by the legislation. Two establishments mentioned imaging within the information booklet, but it was not on the consent form, and one institution had no mention of imaging anywhere in the bequeathal documentation. The list of imaging techniques that could be used was not exhaustive, and 3D imaging modalities were rarely mentioned. One establishment had incorporated imaging into the donation retention tick box such that it was impossible to donate and not consent to images. All bar one establishment that covered imaging made it clear that images would be non‐identifiable. Scottish forms, again, were consistent with each other and used wording that would cover specific forms of imaging “… Imaging may include illustration, photography or digital imaging, X‐ray, ultrasound, MRI or any future imaging method.” The inclusion of “any future imaging method” stretches the limits of informed consent, and there remains a question as to whether this statement covers downstream applications such as 3D printing. If body donation programs are assuming that consent for images does cover 3D printing, work should be done to investigate if potential donors interpret the phrasing around image consent in the same way. When it came to what processes the donations would be exposed to almost all were silent, with only four using the terms “freezing” or “embalmed,” though no further details were provided.

Finally, only seven stated that there was an institutional review panel or formal decision‐making process used to determine further use of a donation (e.g., use in a specific research project within the confines of consent given). Furthermore, only three clarified the role of an institutional ethics committee as part of this process. As per best practices recommendation from the American Association for Anatomy (Balta et al. 2025), there should be documented processes with appropriate oversight established within institutions to review and approve research related to body donors.

### 3.3 Section 3—Consent Requirements

In addition to the earlier list of medical conditions restricting donation, there were other requirements placed on donors for their consent to be recognized (Table 4). Given that many body donation programs are only open to adult donors, age restrictions are to be expected. However, there was a variety of minimum age restrictions across the information packs including 17 years of age (*n* = 3), 18 years (*n* = 5), 21 years (*n* = 1), and even a statement from one institution that they do not accept bequeathals from people under the age of 50. There was also one program that has an authorisation process for donors under the age of 18 (two witness signatures required), and no lower age limit. There were no upper age restrictions in any forms, with six places explicitly stating this. Nine institutions made no mention of any age restrictions at all.

All establishments required one witness signature, with many stating that the witness could be NOK; only two forms suggested the witness could not be the NOK, with one of these specifying the witness was also signing to say the donor was not coerced. Three places suggested that it would ideally not be the NOK but would accept their signature if there was no other witness. It is legal practice in the UK that a will has to be signed by two independent witnesses (Baker 2023). A single witness (permitted to be NOK), therefore, is not in alignment with UK requirements for disposal of someone's estate; though we acknowledge the human body holds a unique and peculiar legal position. It should be noted that the witness has a different role in the two legislations. Within the Human Tissue Act (2004) the witness is signing simply to attest the signature of the donor (Part 1, Section 3, subsection 5, Human Tissue Act 2004). In contrast, Section 34 of the Scottish Act, (Human Tissue (Scotland) Act 2006) states that the witness is signing to confirm the signature, *and* that the donor understands the effect of the nomination and is not acting under undue influence.

Lastly, while the use of a representative form for donors who are physically unable to sign is considered valid consent, only six institutions made prospective donors aware of this option.

### 3.4 Section 4—Disposition of Remains

Almost all (*n* = 23) state that donor bodies are cremated at the end of the donation, specifying that the body can be returned to the family for a private funeral on request (Table 5). Only seven establishments said whether they would inform the family when the cremation was taking place, and that the family could request to attend. Two establishments did inform the family of the time of the cremation, although they said it was not possible for the family to attend. Separate from attendance at the cremation, all establishments stated that they held regular memorial services for donors, and in most cases the families are invited to attend these.

When it came to the disposition of retained body parts, information was less transparent. Fifteen establishments made no mention of the disposition method for retained human material after use. Of those that did provide this information, two stated that retained body parts were later incinerated in the same way that an amputated limb would be disposed of, five stated that they were cremated, one establishment stated that the disposition process was “approved by the HTA” with no other details, and one establishment suggested that they were retained forever.

When considering how ashes following cremation would be handled, some forms (*n* = 4) asked the donor to specify if they were to be scattered or collected by a relative. The majority of institutions (*n* = 19) indicated the NOK or executor would be asked to clarify these arrangements after the donation has been accepted. Only one institution did not share any information as to how ashes would be handled following cremation.

### 3.5 Section 5—Accessing the Form and Information

Nearly all (*n* = 23) of the body donation programs had their own webpage on their institution's site, and these could be easily located by a Google search that included “body donation” and the name of the town or city in which the medical school was located. In just over half of cases (*n* = 13) the information and form could be downloaded directly from the website (Table 6). Ten programs required the donor to contact them first before then emailing or posting the forms to them. Only one program had no dedicated web presence aside from having its contact details available on the HTA's website.

Only seven institutions mentioned that the information was available in other formats including larger text and braille. Six stated that the information could be made available in other languages. Of these, only four institutions offered both other formats and languages. The information packs (excluding forms) varied in length from 3 pages to 25, which considering they should generally contain the same information as each other, is a large variance. Documents from 13 of the body donation programs included a date or version number so potential donors could keep track of how information has changed over time, though not always on both the information and form: 10 information packs and 11 consent forms included a date or version number.

Thirteen programs had their information booklet and consent form as two separate documents. In five cases where the information could be downloaded from a website, it was possible to download the consent form and not the information booklet. One program even had the consent form, but not the information available on the website. For establishments that required contact and could then email the documents, six sent the information and consent form as two distinct documents. In cases where the documents were mailed, these were sent in the same envelope. Although several consent forms mentioned the existence of an information booklet and encouraged the reading of it, only two had a tick box on the consent form where the donor had to confirm that they had read and understood the information.

The contact details of each establishment were clear on all information packs and forms, with most places providing a telephone number, email, and postal address. Two institutions also provided a fax number, which does pose the question as to how frequently the bequeathal documents are reviewed and updated. Finally, most (*n* = 19) of the information and consent forms were written in gender‐neutral language, though five used “his/her” or “sir/madam” at various points.

## 4 Discussion

Informed consent is a cornerstone of ethical medical practices, especially in situations involving human participants, as stipulated by the Nuremberg Code (Schütz and Braswell 2023), although some have argued it is impossible to achieve (Boyd 2015) and previous studies have questioned its existence within the body donation programs in the US (Chung and Lehmann 2002; Zealley et al. 2022; Johnson et al. 2023). This study explored the extent to which donation documents from different UK establishments align with previously published research to provide potential donors with sufficient information to make an informed decision. The analysis revealed substantial disparity in the amount of information provided, with documents ranging from 3 to 25 pages. Although commonalities existed, such as mentions of potential rejection of the donation, the inclusion of a list of medical conditions affecting the eligibility for donation, and the disposition of the body via cremation once the donation has been used, there remained many areas of incongruity. A key omission in a quarter of all information packs was guidance on how to withdraw consent once given; donors must be able to say “no” (Kim 2019). Zealley et al. (2022) and Johnson et al. (2023) highlighted the paucity of information on US donor forms sufficient to constitute informed consent. The variability in the depth of information provided, along with the omission of withdrawal information poses a serious ethical concern. The lack of detail in what will happen to the donation and who will have access to it creates what Farsides and Smith (2020) refer to as the “specification problem”; it is hard to understand what is being consented.

The findings suggest a need for standardization of donation information and forms to ensure they meet ethical requirements for informed consent. The HTA in England and Wales and HMIA for Scotland have each provided template documents intended to standardize the consent process for body donation, so it was surprising to find similar levels of heterogeneity as seen in the US (Zealley et al. 2022). This study demonstrates a greater variance in the forms used in England, Wales, and Northern Ireland than in Scotland. While the HMIA's template for the consent form appears to have been broadly adopted with minimal modifications, the forms in the rest of the UK exhibited substantial customization. Forms aside, the information provided alongside the forms varied greatly, irrespective of where in the UK the body donation program was based. It is understandable that processes will vary between body donation programs, but the provision of information relating to these processes should be of a similar level across body donation programs to ensure that it consistently meets the requirement for informed consent to be given. A single, collaboratively produced form and information pack that provides appropriate consistency would be the ideal approach, and we hope this paper will encourage a review of guidelines by regulators, body donation program staff, and potential body donors. Until this can be achieved, we suggest a number of recommendations (at the end of this paper) to address the disparities and provide a more consistent approach to sharing information about body donation which can be adopted by body donation programs across the world.

This study also assessed information and consent forms against criteria related to the accessibility and underlying inclusivity of the documents. Steps to improving inclusivity are crucial to reach a diversity of potential donors that reflect their local communities. Few institutions stated that they could offer donation forms in alternative formats, restricting accessibility for potential donors. Given that language translations and provision of information in appropriate alternative formats are required to meet licensing standards (Standard Consent 1.e‐f of the HTA's Code C (Human Tissue Authority 2023)), it is likely that programs are indeed ready and able to provide alternative formats but have neglected to state this in their information. Further to this, phrases such as “his/her” can be clunky and disrupt the flow of the reader; replacing this with gender‐neutral terms such as “you” or “they” makes the documentation inclusive of all readers and is a simple change to make. It is also important to make information easily accessible, and sharing this on institutional webpages supports this endeavor. Furthermore, information and consent forms should undergo regular review and updates, and version numbers and revision histories should be included so that donors can easily see what has changed since they consented. After all, many donors complete consent forms years or even decades before they may actually donate their body.

Although a donor typically encounters document(s) from only one body donation program, the wide variance among documents raises questions about consistency in meeting ethical obligations. Research on body donation from a donor's perspective, however, is limited. Smith et al. (2022) demonstrated that donors are not as concerned about certain aspects of the process as anatomists may have thought (e.g., donor body parts being stored separately, who accesses the teaching environment, etc.). Indeed, it should be acknowledged that not everyone wishing to donate their body will want to know the details of the process. Research by Bagian et al. (2024) demonstrates that the majority of donors in their study did not feel they needed to know the details of what their body would be used for. Further research should therefore be done to ascertain the levels of detail that potential body donors want to know about the processes their donation will go through, and that information should be available to those that wish to read it.

## 5 Conclusion and Recommendations

This study highlights inconsistencies and omissions in the information given to body donors and their relatives, but it is not the intention of the conclusions to criticize the dedication of personnel working in body donation programs or the relevant regulatory bodies. We recognize that crucial discussions happen every day to address donors' and their families' inquiries, providing information not included in the analyzed documents in a considerate and empathetic way. Ultimately, we recommend developing a single, standardized form and information pack, that is produced collaboratively (between institutions, regulatory bodies, and potential donors) to minimize omissions caused by customization. To this end, we propose 13 recommendations that could be implemented to enhance the informed consent process for body donation internationally.

1. The information booklet should be in the same document as the consent form, so it is not possible to access one without the other.
2. There should be consistency between the information provided and the wording use in the consent form.
3. There should be a tick box on the consent form to confirm that the information has been read and understood.
4. There should be two witness signatories, one may be NOK and the other an independent witness signing to confirm that the donor understands the effect of the donation and is not acting under undue influence. The NOK should be aware of the intention to donate as their cooperation is usually critical in progressing the donation.
5. There should be a consistent age limit for adult donations, congruent with the national legislation.
6. Information should include a description of the process to withdraw consent.
7. The lengthy list of medical conditions should be changed to a clearer statement that there are some medical conditions that, if present at the time of death, will impact the institution's ability to accept a donation; a brief list that avoids medical jargon could be included. Specifications related to manual handling and storage (i.e., height and weight) should still be included.
8. Information and consent forms should be written using inclusive language, and it should be clear that they can be made accessible in a range of formats and languages.
9. Whilst anatomy establishments should keep the list of users broad to enable courses to be developed without modification of consent forms, there should be some consistency across body donation programs as to which occupations should be granted access and who is considered “public.”
10. The information for NOK regarding actions to be taken after the donor's death should be within the document, but able to be removed so that it can be given to the NOK or executor and filed with them.
11. The information should clarify that there is ethical oversight by a committee within the institution to consider further use of a donation (e.g., specific research projects).
12. All bequeathal documents should include version numbers and dates, to support potential donors to identify whether information has been updated since they originally consented.
13. Phrasing around the acquisition and use of images should capture imaging technologies currently existing and the downstream applications of these images, including their use in education, training, and research, and the possibility of 3D printing from images.

## Data Availability

The data that support the findings of this study are available on request from the corresponding author. The data are not publicly available due to privacy or ethical restrictions.
